# Dysregulation of metabolic pathways by carnitine palmitoyl-transferase 1 plays a key role in central nervous system disorders: experimental evidence based on animal models

**DOI:** 10.1038/s41598-020-72638-8

**Published:** 2020-09-24

**Authors:** Michael Sloth Trabjerg, Anne Skøttrup Mørkholt, Jacek Lichota, Michal Krystian Egelund Oklinski, Dennis Christian Andersen, Katrine Jønsson, Kasper Mørk, Marie-Louise Nibelius Skjønnemand, Lona John Kroese, Colin Eliot Jason Pritchard, Ivo Johan Huijbers, Parisa Gazerani, Angelique Corthals, John Dirk Vestergaard Nieland

**Affiliations:** 1 Department of Health Science and Technology, Aalborg University, 9220 Aalborg, Denmark; 2 Mouse Clinic for Cancer and Aging Research, Transgenic Facility, The Netherlands Cancer Institute, 1066 Amsterdam, The Netherlands; 3 Department of Health Technology, The Technical University of Denmark, 2800 Kgs. Lyngby, Denmark; 4 Department of Science, John Jay College of Criminal Justice, City University of New York, New York, NY 10019 USA

**Keywords:** Demyelinating diseases, Motor neuron disease, Neurodegenerative diseases, Experimental models of disease, Preclinical research, Drug development

## Abstract

The etiology of CNS diseases including multiple sclerosis, Parkinson’s disease and amyotrophic lateral sclerosis remains elusive despite decades of research resulting in treatments with only symptomatic effects. In this study, we provide evidence that a metabolic shift from glucose to lipid is a key mechanism in neurodegeneration. We show that, by downregulating the metabolism of lipids through the key molecule carnitine palmitoyl transferase 1 (CPT1), it is possible to reverse or slowdown disease progression in experimental models of autoimmune encephalomyelitis-, SOD1^G93A^ and rotenone models, mimicking these CNS diseases in humans. The effect was seen both when applying a CPT1 blocker or by using a *Cpt1a P479L* mutant mouse strain. Furthermore, we show that diet, epigenetics, and microbiota are key elements in this metabolic shift. Finally, we present a systemic model for understanding the complex etiology of neurodegeneration and how different regulatory systems are interconnected through a central metabolic pathway that becomes deregulated under specific conditions.

## Introduction

In the last decades, the incidence of central nervous system (CNS) diseases including multiple sclerosis (MS), amyotrophic lateral sclerosis (ALS), and Parkinson’s Disease (PD) have been increasing^1–3^. CNS diseases, specifically neurodegenerative diseases, have a pronounced impact on the patient’s quality of life and represent a major economic and emotional burden to both close relatives and society at large. The etiology and pathogenesis of CNS diseases remain elusive. Consequently, few treatment options exist, and no cure is yet available for those aforementioned CNS disorders. Treatments are mainly limited to partial symptomatic relief, but progression and the long-term outcome often remain unimproved.

Though seemingly individual conditions, several CNS diseases share common etiological and pathological pathways involved in onset and progression such as inflammation, oxidative stress, demyelination, dysbiosis in the gut and changes in metabolism^1,3–5^. Moreover, we know that increased levels of low-density lipoprotein cholesterol (LDL), triglycerides and total cholesterol levels, and decreased levels of high-density lipoprotein (HDL) cholesterol are also associated with disease progression^6–8^. Thus, we propose a novel perspective for looking at MS, ALS and PD pathogenesis through a systemic perspective with focus on the role of dysregulated metabolism in these conditions. In MS, several mechanisms of immunoreactivity have been proposed such as molecular mimicry, bystander activation, and altered peptide ligand^1^. However, studies indicate that upregulated lipid metabolism, and changes in the lipid binding protein of myelin could be the trigger of disease leading to the activation, and ultimately dysregulation, of the immune system^9–11^. The activation of immune cells such as microglia, can lead to the production of reactive oxygen species, and thereby oxidative stress^12,13^. Moreover, a shift to lipid metabolism has been found to drive activity of immune cells and inflammation^14,15^. In PD, several mechanisms leading to neurodegeneration such as mitochondrial dysfunction, and oxidative stress are linked to the metabolism of lipids^16^. Furthermore, PD patients have lower serum levels of lipids, which indicates a potential role of metabolic alterations in disease onset, since a higher level of lipids in serum has been found associated with a low prevalence of PD^6^. In ALS, various disease mechanisms causing irreversible damage to motor neurons such as endoplasmic reticulum stress, inflammation, oxidative stress, mitochondrial dysfunction and glutamate excitotoxicity have also been suggested^17^. A higher level of triglycerides have been associated with longer survival rates in ALS patients^18^. In accordance, ALS-mouse models revealed that a decrease in lipids level could lead to disease progression and that lipid metabolism is upregulated prior to disease^8,19,20^. Based on this evidence, our approach is to investigate neurodegenerative, pathological changes in lipid metabolism using in vivo models of MS, ALS, and PD. Specifically, we aimed at investigating the role of a key mitochondrial molecule, carnitine palmitoyl transferase 1 (CPT1), involved in the regulation of both lipids and glucose, and how its activity in the CNS could impact disease development and progression.

To maintain the homeostasis of energy in the brain, a balance between the oxidation of glucose and that of fatty acids is required^21^. Fatty acid oxidation occurs through the uptake of long-chain fatty acids (LCFA), facilitated by fatty acid transport proteins (FATPs)^22^. Following the uptake, LCFA are converted to acyl-CoA by the acyl-CoA synthase activity of the FATPs^21^. Due to the impermeability of the outer mitochondrial membrane to fatty acyl-CoA, a carnitine shuttle, CPT1, is needed to convert acyl-CoA into acyl-carnitine. This is followed by the second step within the inner mitochondrial membrane, where CPT2 reconverts acyl-carnitine into acyl-CoA and carnitine^21^. This underpins the clear-cut function of the mitochondria, and specifically CPT1 and CPT2, to permit β-oxidation of fatty acids, which is necessary for the production of acetyl-CoA used in ATP production by the Krebs cycle^21,23^.

Energy metabolism is crucial to cell and organ function, but metabolic pathways have often been overlooked in studies relating to CNS injuries and diseases. We designed a series of experiments to understand how dysfunctions in metabolic pathways are involved in the progression, etiology, and onset of neurodegeneration with a focus on MS, ALS, and PD. Here, we present evidence of common pathogeneses and consequently provide a target to act on disease modifying mechanisms in addition to control of symptoms. We hypothesize that metabolic dysregulation is the basis for development and progression of several CNS diseases, specifically those leading to neurodegeneration. Specifically, we propose that the dysregulation is—to some degree—a consequence of a shift from glucose to lipid metabolism, which affects several pathways involved in the etiology and progression of CNS diseases. This metabolic shift can be a consequence of several factors: genetic predisposition, environmental factors or most likely, a combination of both. In this study, we have tested our hypothesis by pharmacologically blocking CPT1 with the antagonist, etomoxir, and genetically by producing a rodent model with a *Cpt1a* mutation. The mutation mimics a known human mutation found in the Inuit population, shown to lower the efficiency of CPT1A from 100 to 22%. This study provides further evidence on the role of metabolic alterations and the effect of targeting CPT1 in MS, ALS, and PD through in vivo studies.

## Results

### Pharmacological and genetic inhibition of CPT1 ameliorates experimental autoimmune encephalomyelitis induction and symptoms

**Figure 1:**
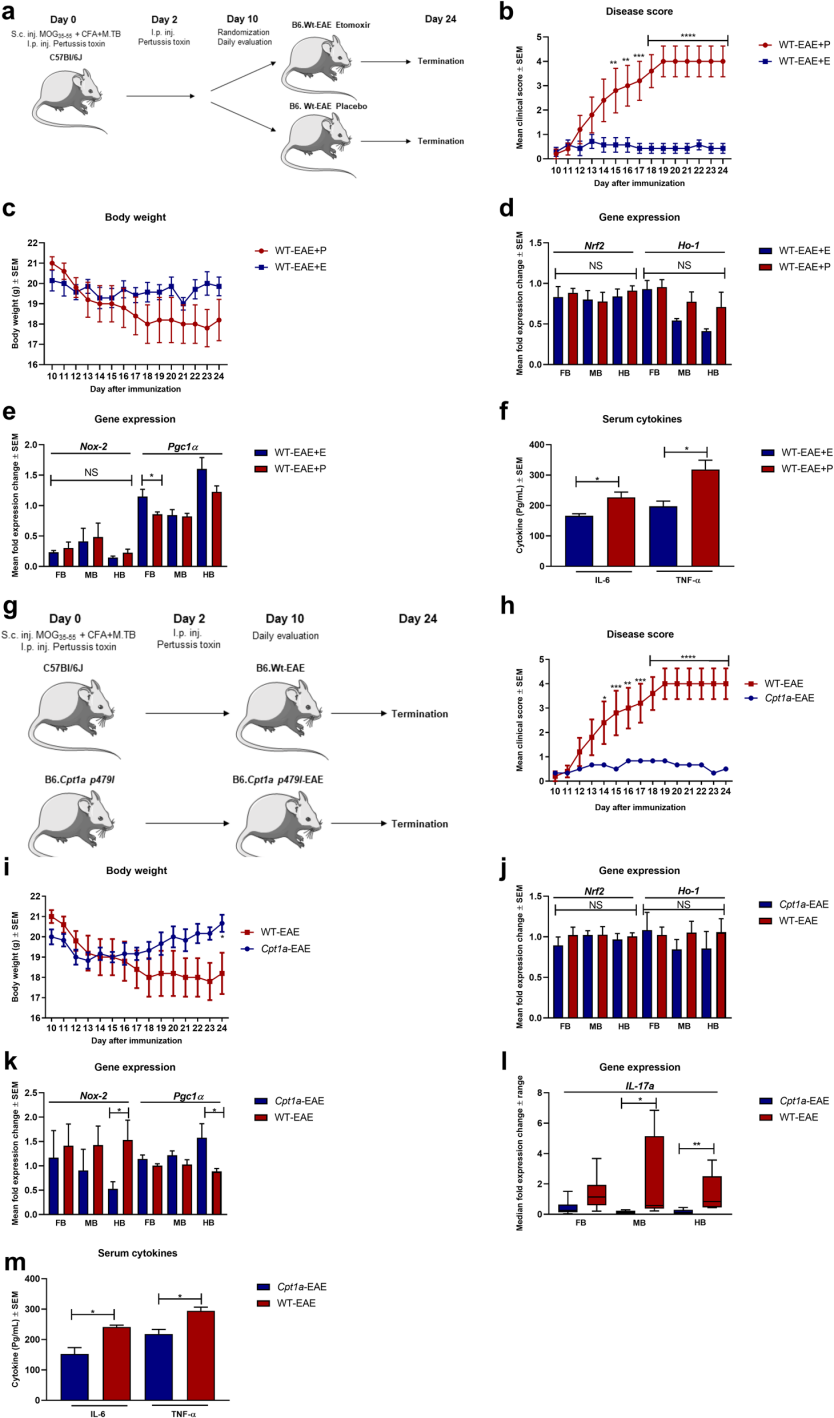
The efficacy of pharmacological and genetic inhibition of CPT1 in EAE models of MS. (**a**) Experimental setup for the EAE-etomoxir study in female C57Bl/6J mice. (**b**) Disease score of WT-EAE-E (n = 7) and WT-EAE (n = 5) mice. (**c**) Body weight of WT-EAE-E (n = 7) and WT-EAE (n = 5) mice. (**d**) Mean fold gene expression change of *IL-17α* in FB, MB and HB of WT-EAE (n = 5) and *Cpt1a*-EAE (n = 6) mice at day 24. (**e**) Mean fold gene expression change of *Nrf2* and *Ho-1* in FB, MB and HB of WT-EAE-E (n = 7) and WT-EAE-P (n = 5) mice at day 24. (**f**) Cytokine level of IL-6 and TNF-α in serum of WT-EAE-E (n = 3) and WT-EAE-P (n = 3) mice at day 24. (**g**) Experimental setup for the *Cpt1a*-EAE study comparing *Cpt1a*-mutant mice with C57Bl/6J female mice. (**h**) Disease score of WT-EAE (n = 5) and *Cpt1a*-EAE (n = 6) mice from day 10. (**i**) Body weight of WT-EAE (n = 5) and *Cpt1a*-EAE (n = 6) mice from day 10. **(j**) Mean fold gene expression change of *Nrf2* and *Ho-1* in FB, MB and HB of WT-EAE (n = 5) and *Cpt1a*-EAE (n = 6) mice at day 24. (**k**) Mean fold gene expression change *Nox-2* and *Pgc1α1* in FB, MB and HB of WT-EAE-E (n = 7) and WT-EAE-P (n = 5) mice at day 24. (**l**) Mean fold gene expression change of *Nox-2* and *Pgc1α1* in FB, MB and HB of WT-EAE (n = 5) and *Cpt1a*-EAE (n = 6) mice at day 24. (**m**) Cytokine level of IL-6 and TNF-α in serum of WT-EAE (n = 3) and *Cpt1a*-EAE (n = 4) mice at day 24. Asterisks indicate the level of statistical significance (*p < 0.05, **p < 0.01, ***p < 0.001, ****p < 0.0001). Abbreviations: *Cpt1a,* carnitine palmitoyl transferase 1a with a p479l mutation; E, etomoxir; EAE, experimental autoimmune encephalomyelitis; WT, wild type; FB, forebrain; MB, midbrain; HB, hindbrain; MOG, Myelin-oligodendrocyte glycoprotein_35–55_ (MOG_35–55_), CFA, Complete Freund's Adjuvant; S.c., Subcutaneous; I.p., Intraperitoneal; Inj., Injection; SEM: standard error of the mean. We acknowledge Servier Medical Art for the mouse illustration, which can be found at: https://smart.servier.com/ with the following license; https://creativecommons.org/licenses/by/3.0/. No changes were made to the drawing.

An increased expression of CPT1A has been demonstrated in MS patients and EAE models^24,25^. Consequently, the effect of pharmacological inhibition of CPT1 by etomoxir was evaluated in the experimental autoimmune encephalomyelitis (EAE) animal model of MS (Fig. 1a). Myelin-oligodendrocyte glycoprotein_35–55_ (MOG_35–55_) immunized C57bl/6J female mice receiving etomoxir (WT-EAE + E) (n = 7) showed a significant decreased mean clinical score compared to the mice receiving placebo treatment (WT-EAE + P) (n = 5) (Fig. 1b) and no decrease in body weight (Fig. 1c). Following the animal experiments, we conducted RT-qPCR to evaluate whether etomoxir changed the gene expression of markers of oxidative stress and mitochondrial function in the fore-, mid-, and hindbrain. Interestingly, we found that etomoxir-treated mice had a tendency towards lower gene expression of *Ho-1* and *Nox-2* in the brain (Fig. 1d,e), although it did not reach statistical significance. However, etomoxir-treated mice had statistical higher expression of *Pgc1α* (Fig. 1e) in the forebrain, indicating amelioration of mitochondrial dysfunction at least in some parts of the brain. To evaluate whether etomoxir treatment had affected the inflammatory response, we evaluated serum levels of IL-6 and TNF-α and found that etomoxir-treated mice had significantly lower levels of both cytokines (Fig. 1f). This indicates that downregulation of CPT1 lipid metabolism affects several key processes in the disease progression of EAE.

To further assess the contribution of CPT1A in the development of EAE, we generated *Cpt1a*-mutated mice^26^ and compared these with WT mice. Both WT-EAE (n = 5) and *Cpt1a*-EAE (n = 6) female mice were immunized with MOG_35–55_ (Fig. 1g). The results showed that the WT-EAE mice presented worsening of mean clinical scores of up to 4, compared to the *Cpt1a*-EAE mice with a mean clinical score of 0.5. The difference between the two groups was statistically significant from day 14 of the experiment and onwards (Fig. 1h). The body weight was also used as a hallmark to evaluate the effect of EAE immunization. The body weight in the group of WT-EAE mice (n = 5) decreased from day 10 to day 24, and was significantly different from the body weight of the *Cpt1a*-EAE mice (n = 6) at the last day of the experiment (Fig. 1i). Based on the clinical effects, we evaluated whether the *Cpt1a*-mutation changed gene expression of markers of oxidative stress, mitochondrial function, and inflammation in the fore-, mid-, and hindbrain of both WT-EAE and *Cpt1a*-EAE mice. The *Cpt1a*-EAE group had lower expression of *Ho-1, and* significantly lower expression of *Nox-2* in the hindbrain (Fig. 1j–k). This indicated that the *Cpt1a* mutation affected oxidative stress. Moreover, the *Cpt1a*-EAE group had significantly higher expression of *Pcg1α* in the hindbrain (Fig. 1k) indicating that the downregulation of CPT1A lipid metabolism resulted in amelioration of mitochondrial dysfunction. The *Cpt1a*-EAE group had significantly lower *IL-17α* expression in the mid-, and hindbrain (Fig. 1l). This was consistent with lower serum levels of IL-6 and TNF- α in serum compared to WT-EAE mice (Fig. 1m). This indicated that downregulation of CPT1A activity was able to diminish inflammation.

### *Cpt1a* mutation provides resistance to high fat diet accelerated EAE induction and symptoms

**Figure 2:**
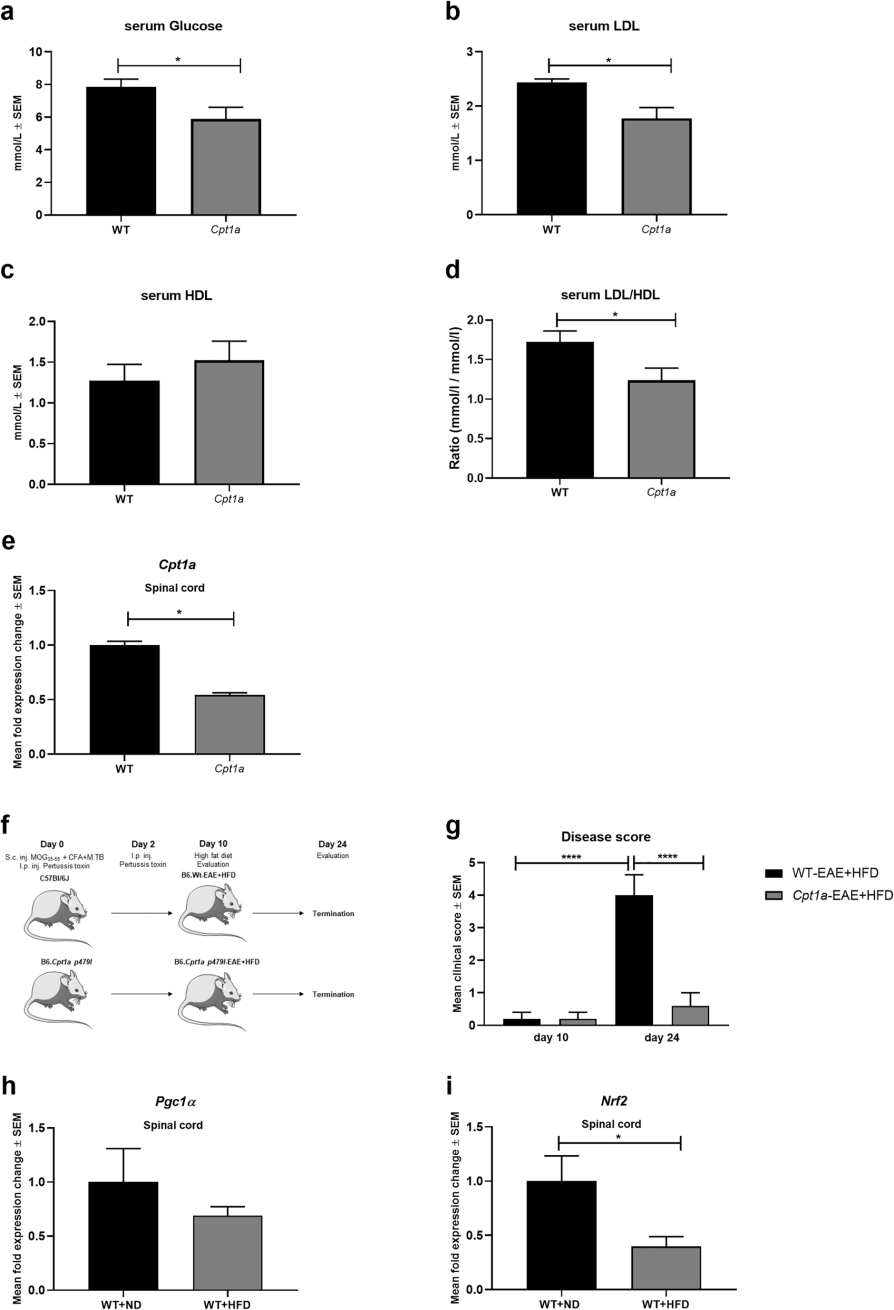
*Cpt1a*-mutated mice have changes in serum metabolites and high fat diet affects disease progression in EAE mouse model, and gene expression in spinal cord. (**a**) Glucose level in serum from *Cpt1a*-mutated male mice (n = 12) and WT mice (n = 15). (**b**) LDL level in serum from *Cpt1a*-mutated male mice (n = 6) and WT mice (n = 5). (**c**) HDL level in serum from *Cpt1a*-mutated male mice (n = 6) and WT mice (n = 5). (**d**) LDL/HDL ratio in serum from *Cpt1a*-mutated male mice (n = 6) and WT mice (n = 5). (**e**) Mean fold gene expression change of *Cpt1a* in spinal cord of female WT- (n = 3) and *Cpt1a*-mutated mice (n = 3). (**f**) Experimental setup for the high fat diet *Cpt1a*-EAE and WT-EAE study comparing the effect of HFD on EAE disease progression. (**g**) Efficacy of high-fat diet on EAE disease severity of WT-EAE (n = 5) and *Cpt1a*-EAE (n = 6) mice from day 10 to day 24. (**h**) Mean fold gene expression change of *Pgc1α* in spinal cord of female WT- (n = 3) and *Cpt1a*-mutated mice (n = 3). (**i**) Mean fold gene expression change of *Nrf2* in spinal cord of female WT-ND (n = 4) and WT-HFD mice (n = 4). Asterisks indicate the level of statistical significance (*p < 0.05). Abbreviations: Cpt1a, carnitine palmitoyl transferase 1a with a p479l mutation; ND, normal diet, HFD, high fat diet. E, etomoxir; P, placebo; EAE, experimental autoimmune encephalomyelitis; MOG, Myelin-oligodendrocyte glycoprotein_35–55_ (MOG_35–55_), CFA, Complete Freund's Adjuvant; S.c., Subcutaneous; I.p., Intraperitoneal; Inj., Injection; WT, wild type. SEM: standard error of the mean. We acknowledge Servier Medical Art for the mouse illustration, which can be found at: https://smart.servier.com/. License; https://creativecommons.org/licenses/by/3.0/. No changes were made to the drawing.

The *Cpt1a*-mutated mouse model resembles the human *CPT1A* mutation found in the Inuit population living in Canada and Greenland. The *CPT1A* nucleotide mutation located at position 1436 C > T predicting a substitution of proline to leucine at codon 479 (*P479L*)^27^ resulting in a residual activity of 22% of the CPT1A protein^28^. In accordance with this, we have found that *Cpt1a*-mutated mice have changes in glucose, LDL and HDL levels (Fig. 2a–d) and that *Cpt1a*-mutated mice have decreased *Cpt1a* expression in the spinal cord (Fig. 2e). High-fat diet (HFD) is associated with increased disease severity of both MS and EAE by changes in metabolism, inflammation and oxidative stress^26^. Specifically HFD has been found to upregulate CPT1A lipid metabolism^29^. Therefore, we investigated the effect of HFD in the EAE model using female *Cpt1a* and WT mice (Fig. 2f). The results showed that the mean clinical scores of the WT-EAE + HFD female mice (n = 5) increased significantly from day 10, at which the mice were subjected to the HFD, until day 24. Additionally, the mean clinical scores were significantly lower for the *Cpt1a*-EAE + HFD mice (n = 6) at day 24 compared to WT-EAE + HFD (Fig. 2g). Interestingly, we have found that female WT mice receiving HFD for 10 weeks have decreased expression of *Nrf2* and *Pgc1α* in the spinal cord compared to WT + ND mice (Fig. 2h–i). The data of low disease induction in the *Cpt1a*-EAE mice found in this study (Fig. 2c–e) support the findings of low prevalence of MS in this northern indigenous population compared to the non-indigenous population in Canada^30,31^.

### *Cpt1a* is epigenetically regulated in the rat blood brain barrier

**Figure 3:**
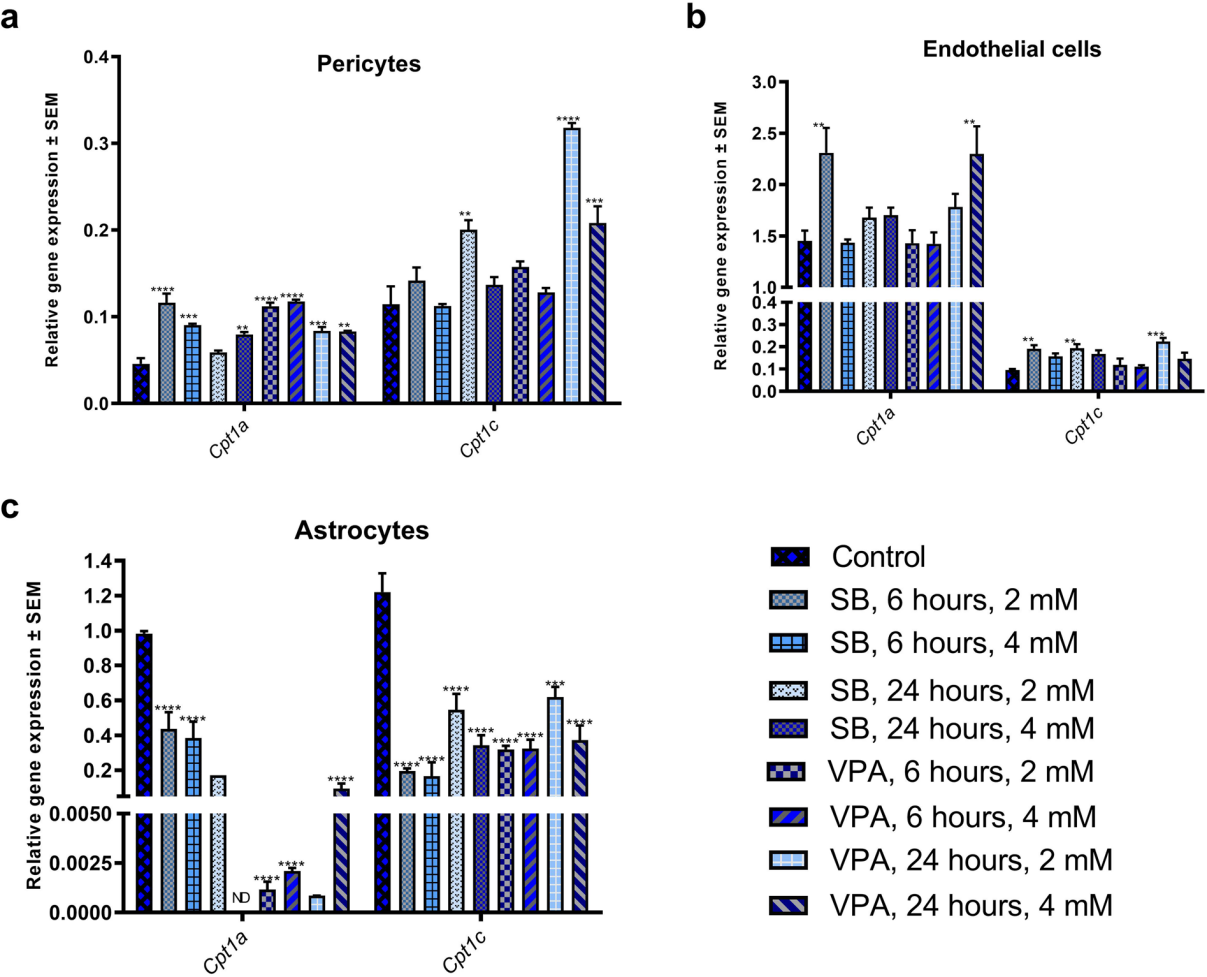
Expression of *Cpt1a* and *Cpt1c* in primary rat BBB-associated cells following treatment with VPA and SB. (**a**) Relative gene expression of *Cpt1a* and *Cpt1c* in pericytes following treatment with VPA and SB. (**b**) Relative gene expression of *Cpt1a* and *Cpt1c* in endothelial cells following treatment with VPA and SB. (**c**) Relative gene expression of *Cpt1a* and *Cpt1c* in astrocytes following treatment with VPA and SB. Data expressed as relative expression to reference genes *β-actin* and *Hprt1* ± SEM. Asterisks indicate the level of statistical significance (**p < 0.01, ***p < 0.001, ****p < 0.0001). Abbreviations: SB, sodium butyrate; VPA, valproic acid. SEM: standard error of the mean.

*Cpt1a* gene is a key regulator of long chain fatty acids metabolism. The transcriptional and post-transcriptional regulation of the Cpt1a in every living organism facilitate adaptation to the changing environment, especially to diet. Recently, Moody et al. provided evidence for epigenetic regulation of *Cpt1a* by DNA methylation and histone modifications in liver of rats fed HFD^32^. The human study of blood samples from the great Dutch famine also showed regulation of *Cpt1a* by DNA methylation^33^. The Blood Brain Barrier (BBB) is the tight epithelium regulating the influx of, among others, metabolites into the brain. Therefore, we used primary BBB-associated cells (endothelium, astrocytes, pericytes) obtained from rats to investigate the effect of two histone deacetylase inhibitors (HDACi), valproic acid (VPA) and sodium butyrate (SB), on the expression of *Cpt1a* gene. *Cpt1a* exhibited significant expression changes in response to both HDACi in the pericytes (Fig. 3a), endothelium (Fig. 3b), and astrocytes (Fig. 3c). As expected, both HDACi led to the increased expression of the *Cpt1a* in pericytes and endothelial cells through augmented histone acetylation. Unexpectedly, expression of *Cpt1a* almost disappeared in the astrocytes. More investigation of the role of histone acetylation levels must be done to understand the precise mechanism of HDACi effect on the *Cpt1a* promoter.

### Pharmacological and genetic inhibition of CPT1 downregulates disease progression in SOD1^G93A^ mice reflected on neurological scores and gene expression

**Figure 4:**
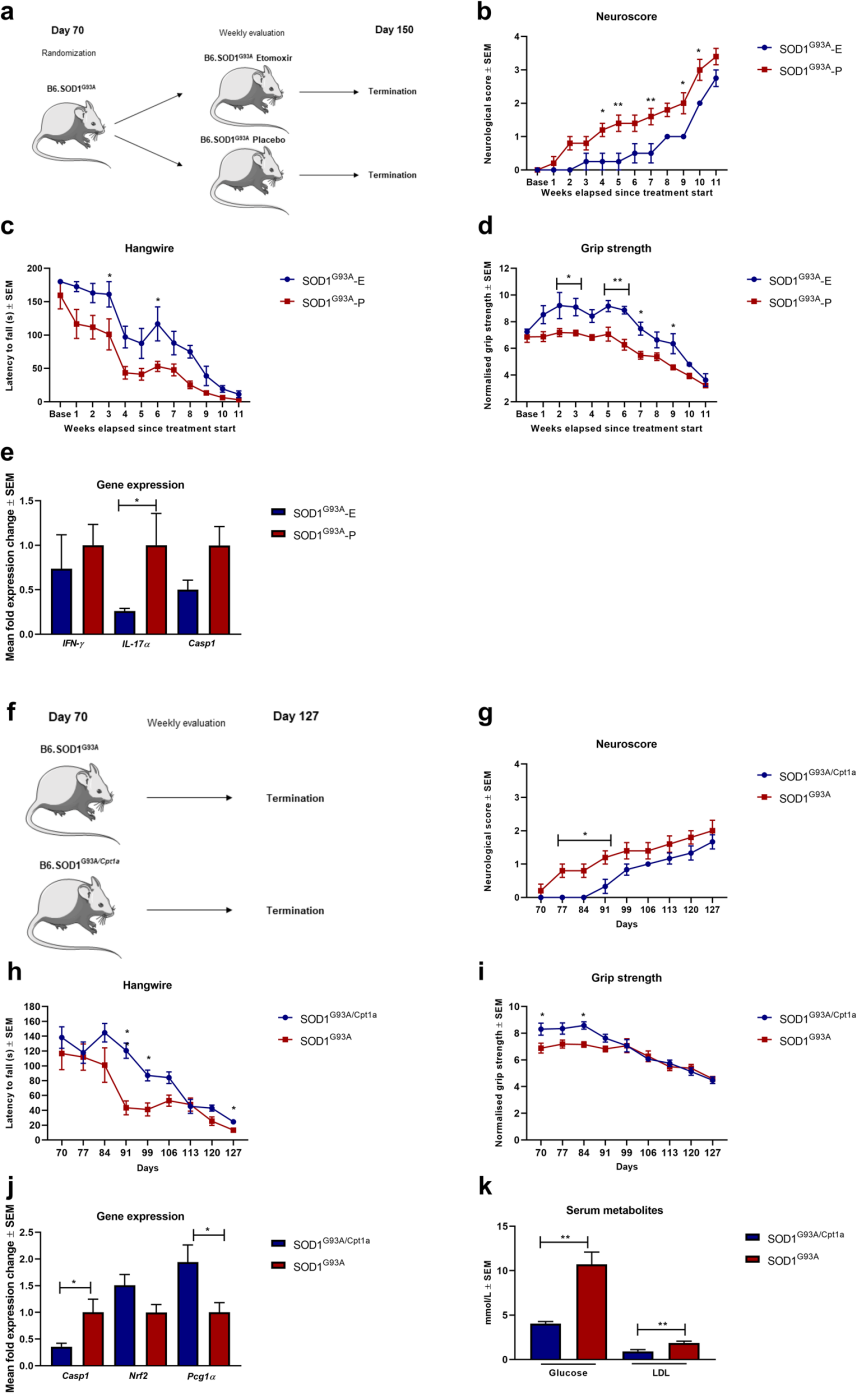
The efficacy of pharmacological and genetic inhibition of CPT1 in SOD1^G93A^ model of ALS. (**a**) Experimental setup for the SOD1^G93A^-etomoxir study using female mice. (**b**) Neuroscore of female SOD1^G93A^-E (n = 4) and SOD1^G93A^-P (n = 5) mice. (**c**) Latency to fall on the hang-wire test of female SOD1^G93A^-E (n = 4) and SOD1^G93A^-P (n = 5) mice. (**d**) Normalized grip strength of female SOD1^G93A^-E (n = 4) and SOD1^G93A^-P (n = 5) mice. (**e**) Mean fold expression change of *Ifn-ϒ*, *Il-17α* and *Casp1* in spinal cord of SOD1^G93A^-E and SOD1^G93A^-P mice (n = 3–4). (**f**) Experimental setup for the SOD1^G93A/Cpt1a^ study comparing heterozygote *Cpt1a* mutation with SOD1^G93A^ female mice without mutation. (**g**) Neuroscore of SOD1^G93A^ (n = 5) and SOD1^G93A/Cpt1a^ (n = 6) mice. (**h**) Latency to fall on the hangwire test of SOD1^G93A^ (n = 5) and SOD1^G93A/Cpt1a^ (n = 6) mice. (**i**) Normalized grip strength of SOD1^G93A^ (n = 5) and SOD1^G93A/Cpt1a^ (n = 6) mice. (**j**) Mean fold expression change of *Casp1*, *Nrf2* and *Pgc1α* in spinal cord of SOD1^G93A^ and SOD1^G93A/Cpt1a^ (n = 3–4). (**k**) Level of glucose and LDL in serum SOD1^G93A^ and SOD1^G93A/Cpt1a^ (n = 4) mice. Asterisks indicate the level of statistical significance (*p < 0.05, **p < 0.01). Abbreviations: SOD1^G93A^, superoxide dismutase 1 glycine 93 changed to alanine; Cpt1a, heterozygote carnitine palmitoyl transferase 1a with a p479l mutation; E, etomoxir; P, placebo. ALS: amyotrophic lateral sclerosis. SEM: standard error of the mean. We acknowledge Servier Medical Art for the mouse illustration, which can be found at: https://smart.servier.com/ with the following license; https://creativecommons.org/licenses/by/3.0/. No changes were made to the drawing.

Several studies have shown that both ALS patients and animal models mimicking ALS have a dysregulated metabolism of lipids^19,34^. CPT1A and CPT1B are upregulated in the SOD1 mouse model mimicking familial ALS^20,35^. Therefore, we tested the effect of pharmacological inhibition of CPT1 by etomoxir in the SOD1^G93A^ model. The SOD1^G93A^ female mice (n = 9) were randomized into treatment with either etomoxir (5 mg/kg) (n = 4) or placebo (olive oil) (n = 5) from day 70 (baseline) (Fig. 4a). The SOD1^G93A^ mice receiving etomoxir (SOD1^G93A^-E) showed significantly lower mean neurological score, increased grip strength, and higher latency to fall on the hangwire test compared to mice treated with placebo (SOD1^G93A^-P) (Fig. 4b–d). Following the SOD1^G93A^ etomoxir experiment, we assessed gene expression in the lumbar spinal cord of the animals to evaluate whether the downregulation of CPT1 affected inflammatory markers. We found that etomoxir-treated SOD1^G93A^ mice had a tendency towards lower expression of *Ifn-ϒ*, *Casp1,* and significant lower expression of *IL-17α* (Fig. 4e). This indicated that downregulation of CPT1 reduces inflammation. These findings support previously presented data that dysregulated metabolism is most likely associated with ALS^36^.

To assess the role of CPT1A in the development and progression of ALS, we generated a SOD1^G93A^ mouse model with a heterozygote *Cpt1a P479L* mutation (SOD1^G93A/Cpt1a^) with 66% CPT1A activity compared to the wildtype CPT1A (Fig. 4f). The development and progression of disease in the female SOD1^G93A/Cpt1a^ mice (n = 6) were compared to SOD1^G93A^ mice (n = 5) from day 70. The SOD1^G93A/Cpt1a^ mice had a significantly lower disease score, higher normalized grip strength and a higher latency to fall on the hang-wire test compared to SOD1^G93A^ mice in the early symptomatic stage of the disease but not later (Fig. 4g–i). Following termination of the animal experiments, we assessed changes in gene expression in the lumbar spinal cord to evaluate inflammation, oxidative stress and mitochondrial functioning. We found that SOD1^G93A/Cpt1a^ mice had significantly lower *Casp1* expression, higher *Nrf2* and significantly higher *Pgc1α* expression (Fig. 4j). This indicates that SOD1^G93A/Cpt1a^ had lower inflammation, better oxidative stress defense and mitochondrial biogenesis. Further, we assessed the levels of glucose and LDL in the serum of SOD1^G93A/Cpt1a^ mice and compared these to SOD1^G93A^ mice to assess whether the *Cpt1a* mutation affected the metabolism. Interestingly, we found that SOD1^G93A/Cpt1a^ had significantly lower levels of glucose and LDL (Fig. 4k). This indicates that CPT1A lipid metabolism plays a role in the development and progression of the disease.

### Inhibition of CPT1 and CPT1A reverses disease progression in a chronic rotenone animal model mimicking Parkinson’s disease

**Figure 5:**
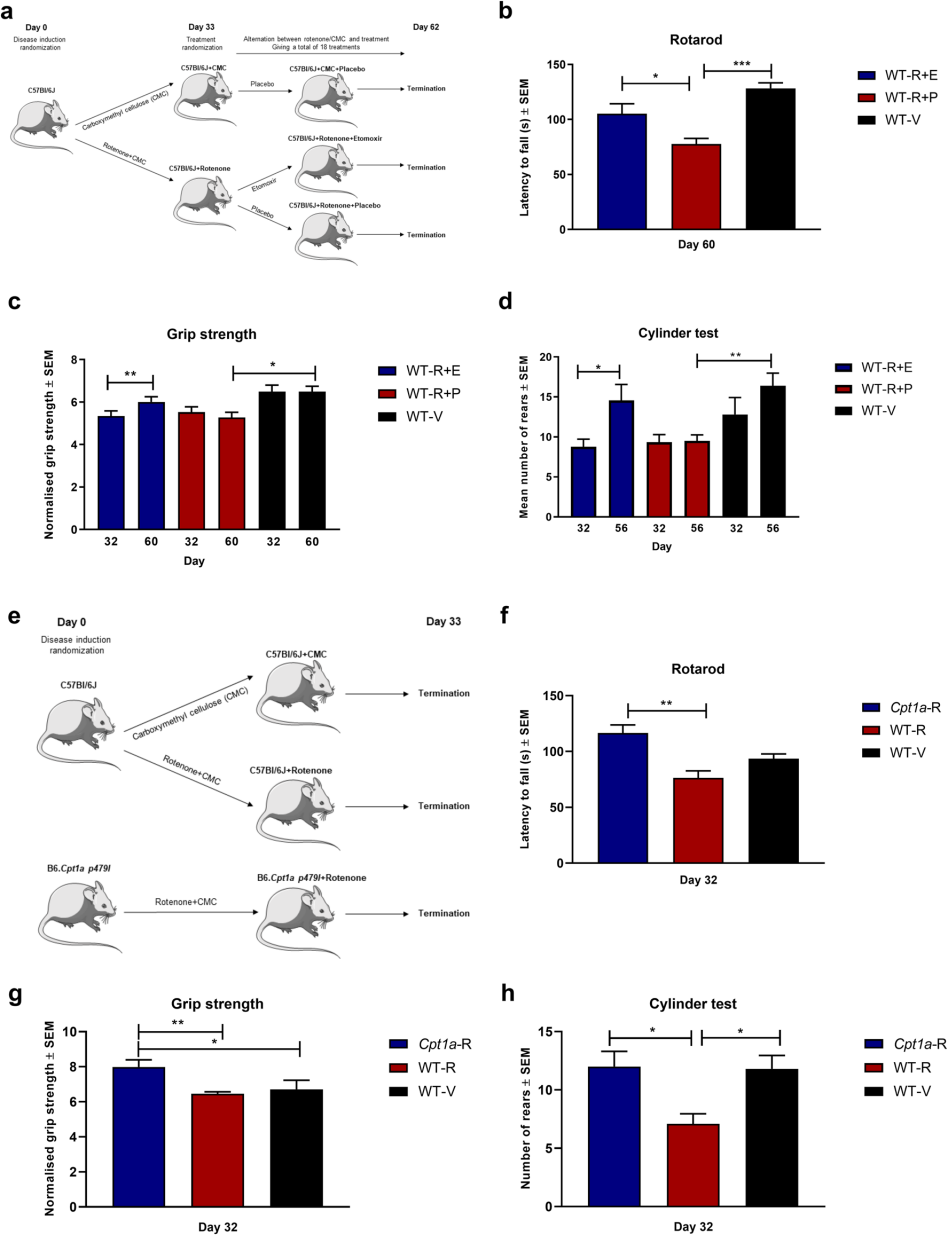
The efficacy of pharmacological and genetic inhibition of CPT1 in rotenone models of PD. (**a**) Experimental setup for the chronic rotenone mouse model treated with etomoxir using male C57Bl/6J mice. (**b**) Latency to fall of the rotarod at day 60 for WT-R-E (n = 9), WT-R-P (n = 9) and WT-V (n = 5). (**c**) Normalized grip strength for WT-R-E (n = 9), WT-R-P (n = 9) and WT-V (n = 5) at day 32 before treatment and at day 60. (**d**) Number of rears in the cylinder test for WT-R-E (n = 9), WT-R-P (n = 6) and WT-V (n = 5) at day 32 before treatment and at day 56. (**e**) Experimental setup for the chronic rotenone mouse model using *Cpt1a*-mutated mice and male C57Bl/6J mice. (**f**) Latency to fall of the rotarod at day 32 for *Cpt1a*-R (n = 7), WT-R-P (n = 10) and WT-V (n = 5). (**g**) Normalized grip strength at day 32 for *Cpt1a*-R (n = 7), WT-R-P (n = 10) and WT-V (n = 5). (**h**) Number of rears in the cylinder test at day 32 for *Cpt1a-R* (n = 5), WT-R-P (n = 10) and WT-V (n = 5). Asterisks indicate the level of statistical significance (*p < 0.05, **p < 0.01). Abbreviations: Cpt1a, carnitine palmitoyl transferase 1a with a p479l mutation; R, rotenone; E, etomoxir; P, placebo; V, vehicle; WT, wild type. PD: Parkinson’s’ Disease. SEM: standard error of the mean. We acknowledge Servier Medical Art for the mouse illustration, which can be found at: https://smart.servier.com/ with the following license https://creativecommons.org/licenses/by/3.0/. No changes were made to the drawing.

Metabolic dysregulation was shown in several studies in both PD patients and animal models of PD (toxic and genetic)^6,37^. Furthermore, studies have found that β-oxidation is upregulated in PD patients^38,39^. Rotenone is a pesticide which blocks the mitochondrial complex 1 and has been shown to upregulate CPT1. Therefore, the effect of pharmacological inhibition of CPT1 by etomoxir was evaluated in a chronic rotenone mouse model using C57Bl/6J male mice (Fig. 5a). Mice were randomized into a rotenone regimen (30 mg/kg daily, n = 20) or vehicle (0.5% Carboxymethylcellulose sodium salt; CMC, n = 5) for 32 days. The vehicle group served as healthy controls. At day 32, the mice were evaluated for symptoms. Two mice did not develop symptoms of PD, and was removed before initiation of treatment. The rotenone-induced mice were randomized into treatment with etomoxir (5 mg/kg, WT-R + E, n = 9) or placebo (olive oil, WT-R + P, n = 9) (Fig. 5a). The mice receiving CMC were treated with placebo (WT-V). The mice received 18 days of treatment over a period of 30 days (Fig. 5a). The WT-R + E mice had a significantly higher latency to fall on the rotarod compared to WT-R-P mice at day 60 (Fig. 5b). Moreover, etomoxir-treated mice had a significant increase in normalized grip strength compared to day 32 (Fig. 5c) and sensorimotor function in the cylinder test (Fig. 5d). These findings indicate that inhibition of CPT1 could restore motor- and sensorimotor function in the rotenone-induced PD-like C57Bl/6J mouse model.

To investigate whether CPT1A activity contributed to development of PD, we compared the *Cpt1a*-mutated male mice with WT male mice for sensitivity to chronic rotenone induced disease (Fig. 5e). *Cpt1a*-mutated mice (*Cpt1a*-R, n = 7) and wildtype C57Bl/6J mice (WT-R, n = 10) received rotenone for 32 days (Fig. 5e). Further, wildtype C57Bl/6J mice received treatment with vehicle (WT-v, n = 5) for 32 days. The vehicle group served as healthy controls. At day 32, *Cpt1a*-R mice had a significantly higher latency to fall on the rotarod (Fig. 5f), a significantly higher normalized grip strength (Fig. 5g), and a significantly higher sensorimotor function in the cylinder test (Fig. 5h) compared to WT-R mice. This finding highlights that *Cpt1a*-mutated mice, which have a down-regulated lipid metabolism, were resistant to rotenone-induced PD. However, more mechanistic studies are warranted.

### *Cpt1a*-mutated mice have alternations in the gut microbiome

**Figure 6:**
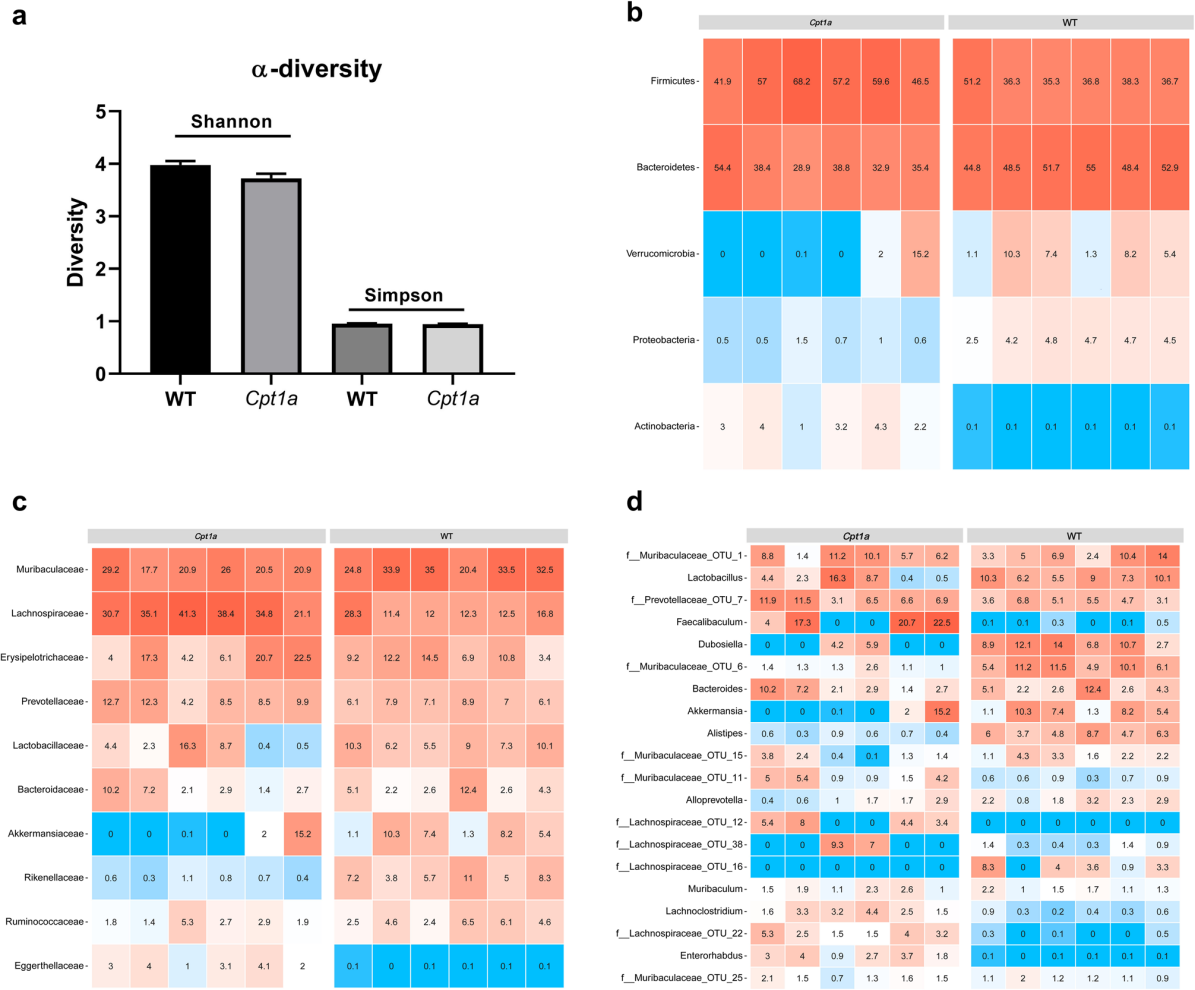
*Cpt1a*-mutated mice have changes in their gut microbiota compared to WT mice. (**a**) Shannon and Simpson α-diversity measures in male *Cpt1a*-mutated (n = 6) and WT mice (n = 6). (**b**) The five most abundant bacterial communities at *phyla* level in male *Cpt1a*-mutated (n = 6) and WT mice (n = 6). (**c**) The ten most abundant bacterial communities at *family* level in male *Cpt1a*-mutated (n = 6) and WT mice (n = 6). (**d**) The 20 most abundant bacterial communities at *genus* level in male *Cpt1a*-mutated (n = 6) and WT mice (n = 6). Heat maps illustrate the relative abundance of the bacterial communities. Abbreviations: *Cpt1a*, carnitine palmitoyl transferase 1a with a p479l mutation; WT, wild type.

The role of gut microbiota in the development and progression of CNS diseases has been intensively studied in the last decade^40^. Based on our findings in the EAE (Fig. 1), SOD1^G93A^ (Fig. 4) and chronic rotenone mouse models (Fig. 5), were *Cpt1a*-mutated mice showed resistance towards disease induction- and progression. We hypothesized that the *Cpt1a p479l* mutation might have affected the gut microbiota composition. To test this hypothesis, 16S rRNA sequencing were performed on fecal samples from male *Cpt1a p479l* mutated mice (n = 6) and male C57Bl/6J mice (n = 6). First, we assessed α-diversity using Shannon and Simpson index and found no differences between the groups (Fig. 6a). Next, we constructed heat maps at the phylum-, family- and genus level (Fig. 6b–d). At the phylum level, the 5 most abundant bacterial communities were *firmicutes*, *bacteroidetes*, *verrucomicrobia*, *proteobacteria* and *actinobacteria* (Fig. 6b). Interestingly, the abundance of *verrucomicrobia* and *proteobacteria* were remarkably low in the *Cpt1a*-mutated- compared to the WT mice, where *actinobacteria* were more prevalent. Following this, we constructed a heat map at the family level illustrating the 10 most abundant communities (Fig. 6c). *Lachnospiraceae* was remarkably more abundant in the *Cpt1a*-mutated mice, whereas *akkermansiaceae*- and *rikenellaceae* were less abundant. Subsequently, we assessed the 20 most prevalent bacterial communities at the genus level (Fig. 6d). Interestingly, the *Cpt1a*-mutated mice had higher abundance of *faecalibaculum*- and lower abundance of *akkermansia*-, *alistepes* communities compared to WT mice (Fig. 6d). Following the heap map analyses, we assessed significant differences at the genus level between *Cpt1a*-mutated- and WT mice and found 132 communities with significant differences between the two groups (Supplementary Table 1). This is the first time that CPT1A activity has been shown to affect the gut microbiota composition.

## Discussion

Neurodegenerative diseases such as MS, ALS, and PD, have a complex, and as of yet, unresolved origin and pathogenesis. As a result, only treatments that address specific symptoms exists at the moment. Here, we have presented that MS, ALS, and PD are characterized by local, and systemic changes in metabolism. Based on a CPT1A mutation found in the Inuit population, which seems to confer resistance to neurodegeneration by reducing CPT1A activity by 78%, we decided to test the effect of CPT1A de-activation in neurodegeneration in two ways: by pharmacologically blocking CPT1, and by creating a new *Cpt1a p479l* mice^26^. The findings from this study revealed that the *Cpt1a*-mutated mice are resistant to the development of not only EAE, but also PD and to some extent ALS. In addition, we tested the efficacy of a CPT1-antagonist, etomoxir, on the same animal models mimicking MS, ALS, and PD and revealed that it could reverse disease progression (Figs. 1, 4, 5).

CPT1, in its three isoforms, is regulated by the Peroxisome Proliferator Activated-Receptors (PPARs) in a tissue-dependent manner^41–43^. PPARs are regulators of multiple pathways including glucose- and lipid metabolism and the immune system^44–46^. As heterodimers with retinoid X receptor, they target genes that are involved in the regulation of mitochondrial- and peroxisomal fatty acid oxidation, fatty acid transport, glucose transport and apolipoprotein synthesis and inflammatory enzymes. Furthermore, they regulate oxidants and chemoattractant ligands, differentiation and recruitment of T cells and cell apoptosis among others^47,48^. PPARs have been associated with CNS diseases in humans and in vivo models including MS, EAE, ALS, SOD1^G93A^, PD, and rotenone models^49–53^. Interestingly, several studies have shown that PPAR-α and PPAR-ϒ agonists ameliorate disease or disease progression in EAE-, rotenone-, and the SOD1^G93A^ mouse models through mechanisms such as decreased demyelination, inhibition of Th1 differentiation and IFN-ϒ production^54–56^. Moreover, PPAR-α- and ϒ agonists decrease oxidative stress and increase mitochondrial biogenesis through *Pgc1α* in these models^52,57^. Etomoxir, our CPT1-antagonist, has been shown to upregulate PPAR-α^58^ and PPAR-ϒ^59^, which could explain the positive clinical effects seen in the EAE- (Fig. 1), SOD1^G93A^ (Fig. 4) and rotenone (Fig. 5) models following treatment. Furthermore, *Pgc1α* was upregulated following etomoxir treatment (Fig. 1e) and *Cpt1a* mutation (Fig. 1k, 4j) indicating that downregulation of the metabolism of lipids by CPT1 has a positive effect through PGC1α.

Inhibition of CPT1 by etomoxir has been shown to downregulate the production of reactive oxygen species^60^. We found a tendency towards a decreased gene expression of *Nox-2* and *Ho-1* in the hindbrain in the EAE models following downregulation of CPT1, and CPT1A (Fig. 1d–e,k). *Nox-2* and *Ho-1* have previously been found upregulated in MS, and EAE^61,62^. We also found, a tendency towards, upregulation of *Nrf2,* previously shown to protect against oxidative stress and correlated with disease activity in SOD1^G93A^ model, in the SOD1^G93A/Cpt1a^ mice (Fig. 4j)^63^.

We have also previously shown that blocking CPT1 using etomoxir reduces demyelination, and increase apoptosis of MOG specific T-cells, decreases production of the inflammatory cytokine IL-17, and decrease production of IL-6^64,65^. We found that *Il-17α* expression was significantly downregulated in the mid-, and hindbrain of EAE-*Cpt1a* mice (Fig. 1i) and SOD1^G93A^ mice following etomoxir treatment (Fig. 4e). This indicates that downregulation of the metabolism of lipids through CPT1 results in a dampening of inflammation, which is supported by lower IL-6 and TNF-α levels in serum from the EAE experiments (Fig. 1f,m) and lower inflammatory gene expression in the spinal cord from the SOD1^G93A^ experiments (Fig. 4e,j). Several studies have shown that metabolism plays a central role in the regulation of immune cells^14,64,66^. Some studies involved the use of etomoxir which was shown to downregulate the inflammatory properties of classical dendritic cells (DC) and increase insulin sensitivity by upregulating the metabolism of glucose through GLUT4^15,67,68^. Correspondingly, we found that *Cpt1a*-mutated mice presented with changes in serum glucose, and lipoproteins in serum indicating changes in metabolism (Fig. 2a–d). These changes were most profound in the disease progression in the SOD1^G93A/Cpt1a^ study (Fig. 4k).

Multiple factors can upregulate the metabolism of lipids through CPT1, such as a diet rich in high saturated fat and refined sugars. Multiple studies have found that HFD increases disease activity in EAE, ALS, and PD in vivo models^69,70^. Remarkably, here we found that *Cpt1a*-EAE mice receiving HFD had significantly lower disease activity compared to WT-EAE mice receiving HFD (Fig. 2g). We hypothesized that this severe disease progression could be due to several factors such as dysregulated mitochondrial function (Figs. 1k, 2h), decreased oxidative defense mechanisms (Figs. 1k, 2i), and inflammation (Fig. 2i) in WT-EAE mice receiving HFD. Another factor that could upregulate CPT1 lipid metabolism might be chronic hyperactivation of the HPA-axis leading to high levels of circulating corticosteroids, which has been reported for MS, ALS and PD^71–73^. The chronic hyperactivation of the HPA-axis results in glucocorticoid receptor resistance and runaway inflammation due to the failure of the negative glucocorticoid feedback mechanism^74^. Furthermore, dysregulation of the HPA-axis results in diminished secretion of insulin, and insulin resistance, resulting in a shift from glucose metabolism to lipid metabolism. In turns, this leads to more inflammation and catabolism of nearby lipids for energy gain^75^.

Our data also indicates that *Cpt1a* expression also regulates the function of the endothelial cells of the blood brain barrier (BBB). The BBB is crucial for healthy brain function, but remains a big challenge in drug delivery^76^. Many neurodegenerative diseases such as MS, ALS, and PD exhibit compromised BBB. The etiology of the leaky BBB is multifactorial and remains very partially understood. Here, we showed that the expression of *Cpt1a* in response to commonly prescribed drugs, VPA, and another HDACi inhibitor SB, affects BBB-associated cells (Fig. 3). *Cpt1a* expression increases significantly in pericytes and endothelial cells in response to treatment with VPA and SB, which is likely to lead to an increase in lipid metabolism at the blood–brain transition zone (Fig. 3a,b). Astrocytes, however, show different *Cpt1a* expression profiles depending on environmental and treatment cues, which might indicate that epigenetic mechanisms could be cell-specific (Fig. 3c). Recently, changes in the *Cpt1a* promoter epigenetic profile were reported in the livers of rats exposed to HFD, which supports the possibility that lipid metabolism can also have influence on the function and integrity of BBB^32^. Changes in histone H3 methylation significantly affected binding of the transcription factor PPARα to the *Cpt1a* promoter^32^. Moreover, acute hyperglycemia—due to an imbalanced glucose/lipid metabolism—has been shown to cause persistent epigenetic changes, including changes to *Cpt1a*^77^. These data, together with the data presented here, indicate that *Cpt1a* does respond to metabolic state through epigenetic processes^32^.

Our study has also shown that mice with a *Cpt1a* mutation exhibited a shift in their microbiota. The gut microbiota has been correlated with the development or protection from CNS diseases and with the modulation of the HPA-axis response from early life^78,79^. Furthermore, stimulates the intestinal gluconeogenesis, modulates glucose- and lipid metabolism, and plays a role in insulin resistance and inflammation^80^. Transplantation of feces from patients with neurodegenerative diseases exacerbates disease severity in animal models mimicking these diseases^81,82^. This indicates that the gut microbiome affects the CNS through involvement of systemic mechanisms. Our data confirms this link between the microbiota and the metabolism by showing that the de-activation of CPT1A modulates the gut microbiota (Fig. 6b–d). Interestingly, we found that several of the significantly changed bacterial communities between *Cpt1a*-mutated mice and WT mice have been reported to play a role in CNS diseases (Supplementary Table 1). *Alistipes* communities have been found significantly increased in humans with mood disorders^83^ and were correlated with the volatile fatty acids and cortisol levels^84^. Further, a higher abundance of *Alistipes* has been associated with type 2 diabetes^85^. *Lachnospiraceae* communities have been found decreased in CNS diseases and were generally more abundant in the *Cpt1a* mutant mice (Fig. 6c)^86^. Interestingly, *Akkermansia* communities have been found to increase the gut permeability and are correlated with PD disease severity and was less abundant in the *Cpt1a-*mutant mice (Fig. 6c–d)^87^. *Faecalibaculum* communities were significantly more abundant in the *Cpt1a*-mutant mice (Supplementary Table 1) and have been found decreased in multiple CNS diseases^86^. The bacterial genus *Blautia* has been found with anti-inflammatory properties and those were significantly higher in the *Cpt1a*-mutant mice (Supplementary Table 1). Interestingly, *Blautia* has been found to be decreased in PD patients^5^. Moreover, *Rikenellaceae* communities were significantly decreased in the *Cpt1a*-mutant mice (Fig. 6c). Interestingly, *Rikenellaceae* has been found elevated following HFD^88^. In addition, the gut microbiota is directly coupled with the diet of the animal or patient. A shift in microbiota has been seen in people under a western diet with high levels of fats and refined carbohydrates^89^. Through our study, we have confirmed that a high fat diet worsens disease severity and progression. The important question remains unanswered on whether the microbiota is changed due to the diet or due to the disease, and whether the disease is induced due to the change in microbiota or due to the diet itself. Our study indicates that it is the former: the CPT1A-mediated lipid metabolism modifies and possibly regulates the microbiota in the gut. Our data also confirms that there is a strong association between microbiota, diet, and disease development or progression. This suggests an important link between these environmental cues and the regulation of gut microbiota.

**Figure 7:**
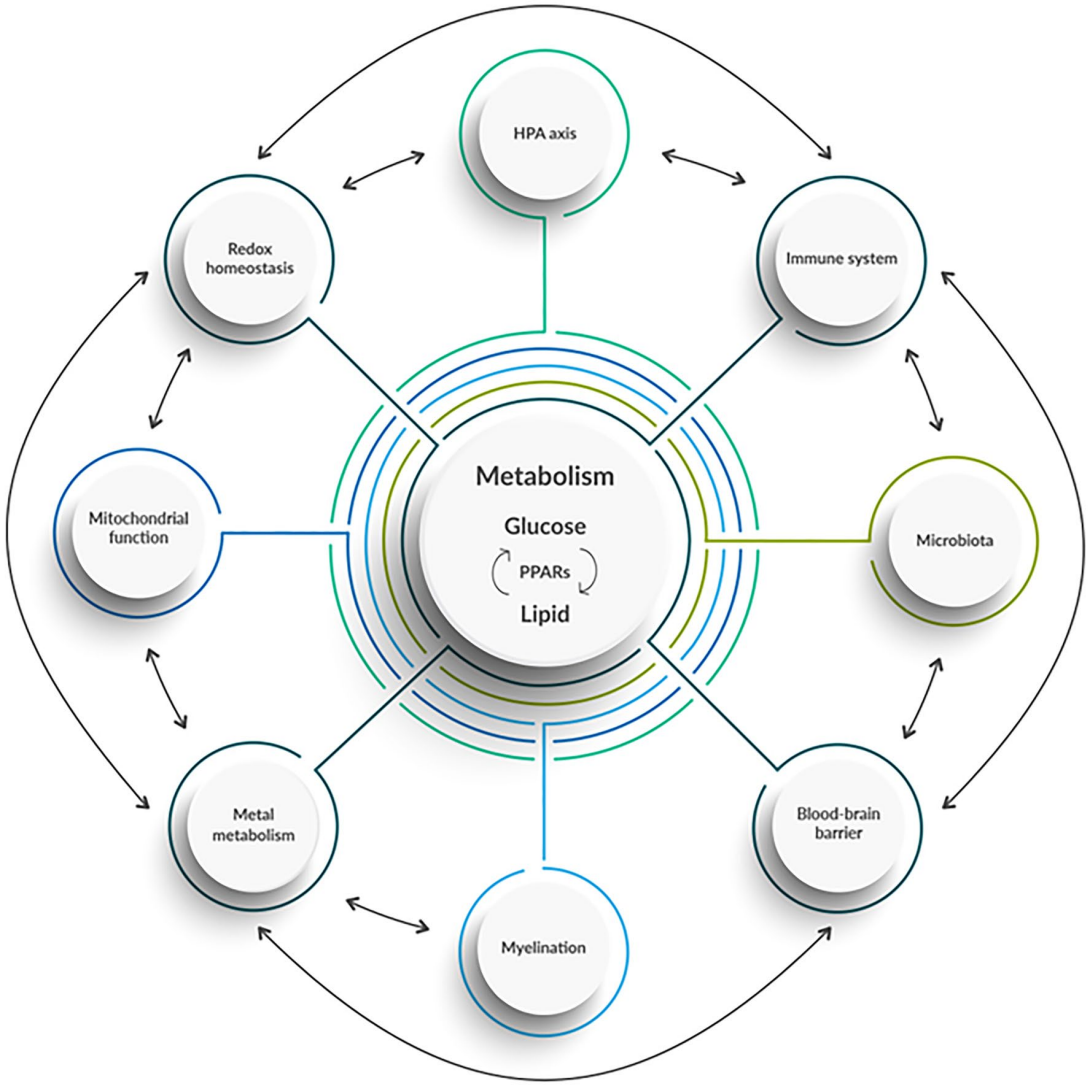
Novel proposed framework of CNS-related disease pathogeneses. We acknowledge Maria Rasmussen for drawing the figure.

Based on the data presented here, we therefore suggest a new model to explain the development and progression of CNS diseases in which an imbalance between the metabolism of lipids and glucose is at the root of neurodegeneration (Fig. 7). A healthy state requires a homeostatic equilibrium between the HPA axis, immune system, microbiota, mitochondrial functioning, metal metabolism, redox state, and glucose-lipid metabolism. Our new model visualizes the possible interplay between these complex processes, and shows that, when imbalanced, they raise the likelihood of CNS disease development. Our study confirms that neurodegeneration is a multifactorial process: no single mechanism seems capable of explaining the complex nature of neurodegeneration in diseases such as MS, ALS, and PD. However, our experiments highlighted a key node in maintaining homeostasis: the activity of CPT1. This is the first time that a common, critical, and underlying pathological mechanism has been established for several neurodegenerative diseases based on experimental data. This model provides an open platform for future experiments and therapeutic targets.

In conclusion, we have provided evidence that CPT1 is a potential key candidate for the regulation of neurodegeneration, acting as the gate-keeper molecule in the homeostasis of lipid and glucose metabolism. Through pharmacological and animal model-based inhibition of CPT1, we have clearly shown that blocking the dysregulated metabolism of lipids offer profound beneficial effects in EAE, SOD1^G93A/Cpt1a^ and rotenone diseases models through decreased inflammation, oxidative stress and increased mitochondrial biogenesis. Importantly, have demonstrated that MS, ALS, and PD, seems to share a common dysregulated metabolism of lipids. We have provided evidence that *Cpt1a* is epigenetically regulated, and that CPT1A lipid metabolism modulates the gut microbiome. Furthermore, we have proposed a new model to understand and target neurodegenerative diseases in future experiments.

## Methods

### Animals

The animal experiments were conducted according to NIH guidelines and were approved by the Danish National Committee for Ethics in Animal Experimentation (Mouse EAE: 2017-15-0201-01240, mouse ALS: 2017-15-0202-00088, mouse PD: 2017-15-0201-01328). All animals were housed in IVC cages and maintained under standardized conditions with a 12 h light–dark cycle and ad libitum access to food and water.

### Generation of *Cpt1a P479L* mice

To examine the role of CPT1A activity, a mouse line (B6J-Cpt1a < em1Nki >) expressing the Inuit mutant allele *Cpt1a P479L* (*Cpt1a* < em1Nki > , MGI number: 5810634) was generated and genotyped as previously described^26^.

### Generation of SOD1^G93A/Cpt1a^ mice

To test the role of *Cpt1a* activity in the *Sod1 g93a* mouse model, mimicking ALS, we generated SOD1^G93A^ mice with a *Cpt1a* point mutation. Briefly, B6.Cg-Tg(SOD1*G93A)1Gur/J^90^ mice were purchased from Jackson Laboratory (Bar Harbor, Main, USA). Mice were acclimated for 3 weeks at 21 °C and kept in a high barrier facility (The Rodent Facility, Aalborg University Hospital). B6.Cg-Tg(SOD1*G93A)1Gur/J males were crossed with homozygote B6J-Cpt1a < em1Nki > female mice. Litters were genotyped using DNA extracted from ear punch tissue using DNA kit (Zymo Research). Mice were genotyped by RT-qPCR using for the expression of the *Sod1 g93a* mutation using primer sequences: (SOD1^G93A^ forward: GGG AAG CTG TTG TCC CAA G, SOD1^G93A^ reverse: CAA GGG GAG GTA AAA GAG AGC, Internal positive control forward: CAC GTG GGC TCC AGC ATT, Internal positive control reverse: TCA CCA GTC ATT TCT GCC TTT G) (TAG Copenhagen) and the previously mentioned primer sequences for *Cpt1a p479l*^26^. RT-qPCR was performed as described below. SOD1^G93A^ female mice with a heterozygote *Cpt1a p479l* mutation (SOD1^G93A/Cpt1a^) (n = 6) was used in subsequent experiment.

### EAE experiments

Sex is known to affect susceptibility to specific diseases and because women are more susceptible to the development of MS, female mice were used in the EAE models^1^.To test the effect of blocking CPT1 by a CPT1-blocker, an EAE model was used^26^. C57BL/6J mice were purchased from Janvier Laboratory (Le Genest-Saint-Isle, France) and were acclimated for 3 weeks at 21 °C in a high barrier before initiating experiment and kept in the Animal Facility at Aalborg University. The 8-week-old C57BL76J female mice (n = 12) were anesthetized with isoflurane and immunized subcutaneously in the base of the tail with 200 μg MOG_35–55_ (Pepmic) emulsified in complete Freund´s adjuvant (CFA) (Becton Dickinson) containing *Mycobacterium tuberculosis* (Becton Dickinson). All mice received an intraperitoneal injection of 500 ng pertussis toxin (Sigma-Aldrich) on the day of immunization and 2 days later. The mice were monitored daily and weighed and scored clinically according to a scale from 0 to 5^26^. The mice were not permitted to lose more than 20% body weight and to go beyond score 4. Mice (n = 7) were treated with etomoxir^25^ (5 mg/kg) (Meta-IQ ApS) or placebo (olive oil) treatment (n = 5) from day 10. To test whether CPT1a activity modulated EAE disease severity, we conduct an animal experiments using female *Cpt1a*-mutated mice. Eight-week-old C57BL76J female mice (n = 5) and *Cpt1a p479l* female mice (n = 6) were induced with EAE as described above. The mice were assessed daily for animal welfare, weighed and scored clinically according to a scale from 0 to 5^26^. The mice were not permitted to lose more than 20% body weight and to go above a clinical score of 4. Further, to test the effect of high fat diet, 8-week-old C57BL76J female mice (n = 5) and *Cpt1a p479l* female mice (n = 6) were induced with EAE as described above and from day 10, the mice received high-fat diet (HFD) (kcal% respectively: protein 20, carbohydrate 20, fat 60, Brogaarden, Denmark; based on D12492 research diets, New Brunswick, USA) throughout the experiments.

### SOD1 G93A experiments

Humans with *SOD1* mutations have an approximately female/male ratio of 1 and because of this females were chosen for the SOD1^G93A^ model^17^.To test whether blocking CPT1 could modulate disease progression in ALS, we used the SOD1 G93A mouse model. B6.Cg-Tg(SOD1*G93A)1Gur/J mice were purchased from Jackson Laboratory (Bar Harbor, Main, USA)^90^. Mice were kept in a high barrier facility (The Animal Facility, Aalborg University). B6.Cg-Tg(SOD1*G93A)1Gur/J male mice were breed with C57Bl/6J female mice. Genotypes were evaluated using DNA extracted from ear tissue punches. RT-qPCR was performed using previously described primer sequences according to protocol^91^. Transgenic SOD1^G93A^ female mice were used for experiments (n = 14). SOD1^G93A^ mice were randomized into treatment with either etomoxir (5 mg/kg dissolved in olive oil) or placebo (olive oil) daily by oral gavage from day 70 (baseline) (Fig. 4a). SOD1^G93A^ mice were evaluated with a neurological score system weekly as previously described^92^ and weighted two times a week. SOD1^G93A^ mice and ALS patients’ losses muscle strength as the disease progresses and therefore muscle strength were assessed weekly using a grip strength meter (Bioseb, France). Grip strength were measured in grams and normalized to weight according to protocol^93^. Furthermore, neurological function and muscle strength were assessed using the hang-wire test as previously described^92,93^. Briefly, mice were put on a wire lid and gently turn upside down. The time until the mice fell down was measured (latency to fall), cutoff time was set to 180 s. Each mice performed three trails and the highest value was used for statistics as previously described^92,94^. Mice were euthanized when they reached a neurological score of 4 or above or if they had a weight loss of 20% or more according to ethical guidelines. The SOD1^G93A/Cpt1a^ mice (n = 6) were evaluated with the same procedures from day 70 until day 127 (Fig. 4f).

### Rotenone experiments

Men have a higher incidence of PD compared to women and, accordingly, we conducted our chronic rotenone model using male mice^3^. To assess whether blockage of CPT1 or CPT1A activity had a role in PD, we used a chronic rotenone mouse model as previously described^95^. Rotenone has been shown to upregulate lipid metabolism through CPT1^96^. Briefly, C57BL/6J male mice were obtained from Janvier Labs (Le Genest-Saint-Isle, France). Mice were acclimated for 3 weeks at 21 °C in a high barrier animal facility. *Cpt1a p479l* mice were generated and breed as previously described^26^. Mice were 9 weeks old when the experiment initiated. Mice were weighted once a week throughout the experiments.

Rotenone is a pesticide and has been used in several animal models of PD^53,95,97,98^. We used a chronic oral gavage, dosing regimen similar to the process reported by others^95,97^. Mice were randomized into a group receiving 30 mg/kg rotenone (Sigma-Aldrich, Cat. 83-79-4) suspended in 0.5% Carboxymethylcellulose sodium salt (CMC) (Sigma-Aldrich, Cat. 9004-32-4) or a group receiving CMC as vehicle daily.. Animals were evaluated by grip strength test as previously described^93^. Rotarod test (Rotamex-5 RotaRod, Columbus Instruments, Columbus, Ohio, USA) with an acceleration from 4 to 40 RPM over 180 s. Mice were acclimatized to the rotarod over three consecutive days. Each mouse was tested 3 times to obtain a mean latency to fall (s). Mice were evaluated for sensorimotor symptoms by the cylinder test as previously described^99^.

For the etomoxir treatment experiment the following groups were used: wildtype mice receiving rotenone (n = 20), and wildtype mice receiving vehicle (n = 5) for 32 days. Animals, which did not show sign of disease defined by the presence of minimum two of the following factors: lower grip strength, lower latency to fall on the rotarod and/or lower number of rears in the cylinder test compared to the healthy control group, was excluded from treatment groups (n = 2) at day 32. At day 33 12 weeks old C57BL/6J mice receiving rotenone were randomized into two groups (Fig. 4a). One receiving etomoxir at 5 mg/kg dissolved in olive oil (n = 9) and another receiving only olive oil as placebo (n = 9). At this point, the daily administration of rotenone was changed to every second day. This intervention was given every second day in between the rotenone and CMC administration, day 33, 35 etc. At day 62, the experiment ended. Thus, making it a total of 43 rotenone administrations spanning along 61 days. The etomoxir intervention group had 18 administrations of etomoxir over a span of 28 days, receiving etomoxir without rotenone intervention from day 50 until 56 and again form day 58 to 60. Animals were evaluated for rotenone induced behavioral deficits at day 32 and day 60, except for the cylinder test which was conducted at day 32 and day 56.

The following groups were used in the *Cpt1a*-mutant experiment; *Cpt1a*-mutant mice receiving rotenone (n = 7), wildtype mice receiving rotenone (n = 10), and wildtype mice receiving vehicle (n = 5) for 32 days (Fig. 4e). Animals were evaluated for rotenone induced behavioral deficits using rotarod, grip strength, and cylinder test at day 32.

### Chronic high fat diet model and *Cpt1a* mutant versus wildtype experiment

10 week old female C57BL/6J mice (n = 8) from our animal facility were randomized into receiving either normal diet (kcal% respectively: protein 29, carbohydrate 65.5, fat 5.5, Brogaarden, Denmark) or HFD (kcal% respectively: protein 20, carbohydrate 20, fat 60, Brogaarden, Denmark; based on D12492 research diets, New Brunswick, USA) for 10 weeks as previously described^100^. After 10 weeks the mice were terminated and spinal cord tissue were quickly harvested and stored at − 80 °C until further analyses. For assessment for differences in serum glucose, LDL, and HDL we used 10 weeks old C57Bl/6J (n = 15), and *Cpt1a* mutant male mice (n = 12). Blood was sampled by retroorbital puncture, and serum was obtained as described below.

### Serum glucose, LDL and HDL analyses

Animals were placed in cages without access to food for 1 h before termination. Blood were obtained from animals and placed at room temperature for minimum 40 min. Afterwards samples were centrifuged at 3500* g* for 15 min and the supernatant was transferred and stored at − 80 °C until further analyses. Serum analyses of glucose- (Crystal Chem, Cat. 81692), LDL- (Crystal Chem, Cat. 79980) and HDL- (Crystal Chem, Cat. 79990) levels were performed using commercial mouse assay kits according to the manufactures protocol. Samples- and standards were run in duplicates and were analyzed using a multiplate reader (PerkinElmer). Concentrations were estimated according to standard curve methods as described in the manufacture’s protocols.

### Gene expression analyses in EAE- and SOD1^G93A^ models

RNA was extracted from the hindbrain, midbrain and frontal lobe for the EAE studies and lumbar spinal cord for the SOD1^G93A^ experiments as previously described^26^. cDNA was synthesized as previously described according to the manufactures protocol^26^. Reverse transcriptase-quantitative polymerase chain reaction (RT-qPCR) was performed as previously described using *β-actin* as housekeeping gene^26^. Samples were run in triplicates for the EAE studies and duplicates for the SOD1^G93A^, *Cpt1a* versus WT and chronic high fat diet model. Following RT-qPCR fold gene expression was determined using 2^−∆∆CT^ method as previously described^26^. *Nox-2*, *Ho-1*, *Nrf2*- and *Pgc1α* primer sequences were as previously described^26^. The following primers were purchased from QIAGEN; *Casp1* (PPM02921E), *Il-17α* (PPM03023A-200), *Ifn-ϒ* (PPM03121A-200).

### ELISA

ELISA was performed using commercial kits for IL-6 (Invitrogen, Cat. 88-7064) and TNF-α (Invitrogen, Cat. 88-7324). Procedures were done according to manufactures protocols. Standards- and samples were run in duplicates. OD values were read at 450 nm using a multiplatereader (PerkinElmer). Calculation of concentrations were done according to manufactures protocol using standard curve methods.

### Microbiota analyses

To assess whether CPT1*a* activity had any impact on gut microbiome community, we investigated changes in the gut microbiota using male *Cpt1a*-muated mice and male wildtype mice. Briefly, fecal samples were collected from 12 week old male *Cpt1a* mice and male wildtype C57Bl/6J mice from Aalborg University animal facility and quickly stored at − 80 °C until further processing. Animals were housed together under identical conditions (bedding, pellets, water and ventilation). 16S rRNA analyses were performed by DNASense APS (Aalborg, Denmark).

### DNA extraction

DNA extraction was performed using the standard protocol for FastDNA Spin kit for Soil (MP Biomedicals) with the following exceptions. 500 L of sample, 480 L Sodium Phosphate Buffer and 120 L MT Buffer were added to a Lysing Matrix E tube. Bead beating was performed at 6 m/s for 4 × 40 s. Gel electrophoresis using Tapestation 2200 and Genomic DNA screentapes (Agilent) was used to validate product size and purity of a subset of DNA extracts. DNA concentration was measured using Qubit dsDNA HS/BR Assay kit (Thermo Scientific).

### Bacterial and Archaeal community analysis targeting 16S V4 rRNA

Bacteria and Archaea, 16S rRNA region V4 sequencing libraries were adapted by a custom protocol established on an Illumina protocol (*Illumina, 2015)* . Extracted DNA (maximum 10 ng) was used as template for Polymerase Chain Reaction amplification of the Bacteria and Archaea, 16S rRNA gene region V4 amplicons. Each Polymerase Chain Reaction reaction (25µL) contained dNTPs (100 M of each), MgSO4 (1.5 mM), Platinum Taq DNA polymerase HF (0.5 U/reaction), Platinum High Fidelity buffer (1X) (Thermo Scientific) and tailed primer mix (400 nM of each forward and reverse primer). Polymerase Chain Reaction was done with the following settings: denaturation at 95 C for 2 min, 30 cycles of amplification (95 °C for 15 s, 55 °C for 15 s, 72° C for 50 s) and a terminal elongation at 72 °C for 5 min. Polymerase Chain Reaction reactions were performed in duplicate for each sample and the duplicates were pooled following Polymerase Chain Reaction. The forward and reverse tailed primers were designed according to^101^ and contain primers targeting the Bacteria and Archaea, 16S rRNA gene region V4: [806RB] GGACTACNVGGGTWTCTAAT and [515FB GTGYCAGCMGCCGCGGTAA]^102^. The amplicon libraries were purified based to the manufactures protocol using Agencourt Ampure XP Beads (Beckman Coulter) with a sample bead to ratio of 5:4. DNA was eluted in 25 µL of nuclease free water (QIAGEN). DNA concentration was confirmed using Qubit dsDNA HS Assay kit (Thermo Scientific). Gel electrophoresis using Tapestation 2200 and D1000/High sensitivity D1000 screentapes (Agilent) was used to validate product size and purity of a subset of sequencing libraries. Sequencing libraries were established from the cleaned amplicon libraries using a second Polymerase Chain Reaction. Each reaction (25 µL) contained PCRBIO HiFi buffer (1x), PCRBIO HiFi Polymerase (1 U/reaction) (PCRBiosystems), adaptor mix (400 nM of each forward and reverse) and up to 10 ng of amplicon library template. Polymerase Chain Reaction was done as follows: denaturation at 95 C for 2 min, 8 cycles of amplification (95 °C for 20 s, 55 °C for 30 s, 72 °C for 60 s) and a final elongation at 72 °C for 5 min. The final sequencing libraries were purified according to the manufactures protocol using Agencourt Ampure XP Beads (Beckman Coulter) with a sample to bead ratio of 5:4. DNA was eluted in 25 µL of nuclease free water (QIAGEN). DNA concentration was quantified using Qubit dsDNA HS Assay kit (Thermo Scientific). Gel electrophoresis using Tapestation 2200 and D1000/High sensitivity D1000 screentapes (Agilent,) was used to validate product size and purity of a subset of sequencing libraries.

The purified libraries were pooled in equiM concentrations and diluted to 2 nM. The samples were sequenced paired-end (2 × 300 bp) on a MiSeq (Illumina, USA). Sequencing was performed using Reagent kit version 3 (Illumina, USA) following the Illumina guidelines for preparing and loading samples on the MiSeq. > 10% PhiX control library was spiked in to overcome low complexity issues often observed with amplicon samples^103^.

### Bioinformatic processing

Forward and reverse reads were trimmed for quality using Trimmomatic v. 0.32^104^ with the settings SLIDINGWINDOW:5:3 and MINLEN: 225 . The trimmed forward and reverse reads were merged using FLASH v. 1.2.7^105^ with the settings -m 10 -M 250. The trimmed reads were dereplicated and formatted for use in the UPARSE workflow^106^. The dereplicated reads were clustered, using the usearch v. 7.0.1090 -cluster_otus command with default settings. OUT abundances were estimated using the usearch v. 7.0.1090 -usearch_global command with -id 0.97 -maxaccepts 0 -maxrejects 0. Taxonomy was assigned using the RDP classifier^107^ as implemented in the parallel_assign_taxonomy_rdp.py script in QIIME^108^, using—confidence 0.8 and the SILVA database, release 132^109^. The results were analysed in R v. 3.5.1 (R Core Team, 2017) through the Rstudio IDE using the ampvis package v.2.4.2^103^.

### Primary cell cultures and treatment

Brain capillary endothelial cells (BCEC) and pericytes were isolated from 2 to 3 weeks old Sprague–Dawley rats. Primary BCECs were cultured in DMEM/Nutrient F-12 Ham (Invitrogen, Cat. 31331-028) with 10% plasma derived bovine serum (First Link, Wolverhampton, United Kingdom, UK), insulin-Transferrin-Selenium (1:100) (Sigma Aldrich I1884), gentamicin sulphate (10 μg/ml), and basic fibroblast growth factor (1 ng/ml) (Roche). To ensure a pure BCEC culture, puromycin (1 μl/ml) (Sigma-Aldrich) was added to the medium.

Primary astrocytes were isolated from neonatal Sprague–Dawley rats and cultured in DMEM (Invitrogen, Cat. 10106-169), fetal calf serum (10%) (Gibco, Cat. 10106-169), and gentamicin sulphate (10 μg/ml) (Sigma-Aldrich, Cat. 17-518Z).

After cell culturing the cells were either used as control cells (n = 3) and/or were treated with and sodium butyrate (2 mM) (SB) (Sigma-Aldrich) or sodium valproate (4 mM) (VPA) (Sigma-Aldrich) for 6- and 24 h (all treatments n = 3). VPA is specific for histone deacetylase (HDAC) class I and IIa, whereas SB inhibits HDAC class IV alongside class I and IIa^110^.

### RNA purification cDNA synthesis

RNA from all samples was purified using AllPrep DNA/RNA Mini kit (QIAGENgen, Cat. 80204) followed by genomic DNA contamination removal using a DNase kit (Thermo Scientific, Cat. EN0525). cDNA was synthesized using Maxima H Minus First Strand cDNA synthesis kit (Thermo Scientific, Cat. K1651).

### RT-qPCR

Reverse transcriptase-quantitative polymerase chain reaction (RT-qPCR) was performed using an AriaMX qPCR machine (Agilent). The following primers were used: *Cpt1a* (QIAGEN), *Cpt1c* (QIAGEN), Hprt1 (5′TGCAGACTTTGCTTTCCTTGGTCA3′, 5′-TGGCCTGTATCCAACACTTCGAG3′) All samples were run in triplicates. The relative expression of the *Cpt1a* was calculated according to previously published methods^111^.

### Statistics

Data was evaluated for normality using Shapiro–wilk test and Q/Q-plots. If normality was present unpaired *t* tests, ordinary one-way ANOVA followed by Tukey’s multiple comparisons post hoc test, ordinary two-way ANOVA followed by Sidak’s multiple comparisons post hoc test, or repeated measures (RM) two-way ANOVA followed by either Tukey’s, Sidak’s or Bonferroni multiple comparisons post hoc test were used to assess statistical significance. If the criteria of normality was not met, a non-parametric Mann–Whitney test was performed. The fold of gene expression was calculated according to previously published methods^111^. All statistics were performed using Graph Pad Prism software. All data are presented as the mean ± SEM, unless otherwise stated. To evaluate possible outliers, a Grubbs test was performed, and outliers were removed. *P*-values of 0.05 were considered significant.

## Supplementary information

Supplementary Information 1

## Data Availability

Data are available upon relevant request to the corresponding author.
